# Molecular Detection of Eukaryotic Diets and Gut Mycobiomes in Two Marine Sediment-Dwelling Worms, *Sipunculus nudus* and *Urechis unicinctus*

**DOI:** 10.1264/jsme2.ME18065

**Published:** 2018-09-29

**Authors:** Yaping Wang, Tiantian Shi, Guoqiang Huang, Jun Gong

**Affiliations:** 1 Yantai Institute of Coastal Zone Research, Chinese Academy of Sciences Yantai China; 2 University of Chinese Academy of Sciences Beijing China; 3 School of Life Sciences, South China Normal University Guangzhou China; 4 Guangxi Institute of Oceanology Beihai 536000 China; 5 School of Marine Sciences, Sun Yat-sen University Zhuhai Campus China

**Keywords:** food source, gut microbiome, life cycle, marine benthos, mycobiome

## Abstract

The present study aimed to reveal the eukaryotic diets of two economically important marine sediment-inhabiting worms, *Sipunculus nudus* (peanut worm) and *Urechis unicinctus* (spoon worm), using clone libraries and phylogenetic analyses of 18S rRNA genes. Fungal rDNA was also targeted and analyzed to reveal mycobiomes. Overall, we detected a wide range of eukaryotic phylotypes associated with the larvae of *S. nudus* and in the gut contents of both worms. These phylotypes included ciliates, diatoms, dinoflagellates, eustigmatophytes, placidids, oomycetes, fungi, nematodes, flatworms, seaweeds, and higher plants. Oomycetes were associated with the planktonic larvae of *S. nudus*. The composition of eukaryotic diets shifted greatly across the larval, juvenile, and adult stages of *S. nudus*, and among different gut sections in *U. unicinctus*, reflecting lifestyle changes during the ontogeny of the peanut worm and progressive digestion in the spoon worm. *Malassezia*-like fungi were prevalent in mycobiomes. *Epicoccum* and *Trichosporon*-related phylotypes dominated mycobiomes associated with larval individuals and in the gut contents of adults, respectively. The gut mycobiome of *S. nudus* was successively characterized through the midgut, aspiratory intestines, hindgut, and rectum as having a high proportion of *Climacodon*-*Rhizochaete*, *Ceriporiopsis*, *Cladosporium*-*Pseudomicrostroma*, and *Malassezia*-related species in the libraries. These results emphasize the dynamics of diets and gut mycobiomes in marine benthic animals.

Animals of the phyla Sipuncula and Annelida are generally diverse and dominant in the marine benthos. Through bioturbation, deposition, and/or suspension feeding, these worms concentrate organic matter and trace elements and regenerate nutrients, thereby playing an important role in sustaining the trophic links of marine benthic food webs (33, 45, 57). A major issue in the functional ecology of these sediment-dwelling worms has been to understand their trophic functions, which include feeding and digestive structures and modes, behavior, and the actual diets ingested and assimilated (33, 45). During the last several decades, biochemical, isotope tracing, and microscopic observation methods revealed that besides bacteria (13, 18, 29), eukaryotic microalgae (mostly diatoms) are important carbon sources for many sediment-dwelling species (*e.g.*, 5, 12, 13) and whole macrofaunal communities in coastal and estuarine sedimentary environments (*e.g.*, 22, 32, 34).

Microscopic observations of the gut contents typically identify dietary eukaryotes at high taxonomic ranks, and may overlook some small, morphologically indistinct, and fragile eukaryotes, potentially resulting in generally low diet breadths (*e.g.*, 12, 61). This is the case for worms living in coastal and estuarine sedimentary environments, at which the diversity of microbial eukaryotes (including microalgae, protozoa, and fungi) is large (24, 66). Eukaryotic diets other than microalgae have yet to be revealed for benthic animals. Ribosomal RNA (rRNA) gene-based molecular methods have recently been increasingly employed to reveal the diets and microbiota of many coastal invertebrate predators with higher classification resolutions. For example, by using the PCR amplification and sequencing of 18S rRNA genes, algal prey or eukaryotic diets in whole guts were identified and quantified for planktonic copepods (49), the larvae of the red rock lobster (50), the adult individuals of marine suspension-feeding bivalves *Mytilus* spp. (46), and mariculture shrimps (16). However, the molecular detection of eukaryotic diets in coastal sediment-dwelling worms has not yet been extensively conducted.

In the present study, we investigated the molecular diversity of eukaryotic diets in two marine species of sediment-dwelling animals, the peanut worm *Sipunculus nudus* Linnaeus, 1766 (Sipuncula, Sipunculidea, Sipunculidae), and the spoon worm *Urechis unicinctus* von Drasche, 1881 (Annelida, Echiura, Urechidae), both of which are economically important in China (43). *S. nudus* is mainly distributed in tropical and subtropical coasts (30) and has been successfully bred artificially in China for decades. The development of *S. nudus* includes three stages: larval, juvenile, and adult. During these successive stages, the food source is markedly altered by changes in behavior and feeding structures and modes, as previously observed using microscopy (61). However, the molecular characterization of gut contents across developmental phases has yet to be performed in detail for this species and other marine worms (29, 33).

*U. unicinctus* is distributed in the coasts of China, Korea, Russia, and Japan (44, 65). Since the artificial breeding of *U. unicinctus* is not that successful, most of these worms are harvested from their natural habitat and available from local seafood markets. Unlike all other deposit-feeding echiurans, adult *Urechis* species are active filtering feeders in that they form a mucous net within a U-shaped burrow through which they draw water by peristaltic contractions to collect food particles (33). The alimentary tract of this species is five-fold longer than its body length, including the midgut, aspiratory intestines, hindgut, and rectum (56), which allows for the sufficient digestion of ingested food and absorption during the gut passage (42, 56). Variations in eukaryotic diets across gut sections need to be investigated in this species and many other marine worms.

The present study investigated compositional variations in major eukaryotic groups across the larval, juvenile, and adult stages of *S. nudus*, and in different gut sections of adult *U. unicinctus*. We also examined the diversity of and changes in the community composition and structure of gut fungi (gut mycobiomes) for the following reasons: 1) among eukaryotes, fungi represent a highly specialized ecological group that may mediate the transformation of non-living organic matter, thereby enhancing nutrient uptake in the anaerobic gut environment (1, 6, 29); 2) sipunculans and echiurans both have markedly longer gut residence times (>1 d) than other worms (mostly between 0.5 and 2 h), which provide longer periods for food caching and re-ingestion. Furthermore, their guts have mildly alkaline pH values, resulting in high digestive efficiencies in extracting organic matter in their diets (33); 3) gut fungi have been isolated from a few marine invertebrates, such as irregular sea urchins (60) and crabs (47, 52), but have rarely been studied in coastal marine benthic invertebrates using culture-independent approaches. The hypotheses that developmental stages and locations in the long alimentary tract affect gut fungal diversity have yet to be tested.

## Materials and Methods

### Specimens and gut content collection

The test organisms of *S. nudus* were originally collected from the coast of Beihai (Beibu Gulf, South China Sea), Guangxi, southern China. The artificial breeding of peanut worms has been successfully conducted in China for a decade; therefore, sampling of the different life stages of this species was easily performed at a breeding base affiliated with the Guangxi Institute of Oceanology in 2014. Three stages of the peanut worm (referred to as SN-l, SN-j, and SN-a) were collected: planktotrophic pelagosphera larvae (body length 0.3–0.4 cm), sediment-dwelling juveniles (2.5–4.0 cm), and adults (10–15 cm). Larvae were obtained from an indoor nursery. Despite a mixed solution of microalgal species being routinely supplied as the main food source to larvae, other unidentified protists/microeukaryotes in the system may have acted as prey. Juveniles were maintained in man-made ponds with nutrient enrichment, periodical water exchange, and amendments with a pelleted artificial diet. The habitat of the adults was completely natural, sediments of tidal flats with a sand content of greater than 70%. Since the gut contents of tiny larvae are difficult to isolate in practice, we assessed the whole larval body instead of dissecting gut tissues.

*U. unicinctus* specimens used in the present study were collected directly from the natural environment, a sandy beach at Yantai (Yellow Sea), Shandong, northern China in August 2014. Living worms were maintained in containers with clean seawater for approximately 4 h before being transported to the laboratory for dissection. Six imaginal individuals (body lengths of approximately 15–30 cm) were randomly selected and taken to the laboratory for dissection. The midgut, aspiratory intestines, hindgut, and rectum (referred to as UU-m, UU-a, UU-h, and UU-r, respectively) were sectioned and pooled in order to examine differences in eukaryotic and fungal diversities among these sections.

The body surfaces of the organisms were repeatedly washed with sterilized seawater and 75% (v/v) ethanol solution before dissection. All dissecting tools were autoclaved before use. Gut sections were sliced into pieces, flushed with sterile seawater, and centrifuged and re-suspended repeatedly in order to discard large pieces of tissue and sediment gravel. The suspension solution containing fine intestinal contents was filtered onto polycarbonate membranes with a pore size of 0.22 μm (47 mm in diameter; Millipore, Burlington, MA, USA), and then placed into cryotubes and stored at −80°C for further processing.

### DNA extraction, PCR amplification, cloning, and sequencing

The extraction and purification of DNA were performed using the FastDNA spin kit (MP Biomedical, Santa Ana, CA, USA) according to the manufacturer’s instructions. The quality of extracted DNA was assessed using gel electrophoresis (1% agarose gels) and quantified using a Nanodrop 2000c spectrophotometer (ThermoFisher, Waltham, MA, USA).

In order to inspect eukaryotic diversity in the gut contents of deposit-feeding marine worms, 18S rRNA genes were PCR amplified using extracted DNA as the template and the eukaryote-specific primers EukA and EukB (48). The PCR program was as follows: 94°C for 3 min, and 35 cycles of 94°C for 1 min, 55°C for 1 min, and 72°C for 2.5 min, with a final extension at 72°C for 10 min. Since no PCR product bands were detected via gel electrophoresis, a nested PCR strategy was applied: 1 μL of first-round PCR product solution was used as a template in second-round PCR amplification using the eukaryotic-specific primer sets Euk1A and Euk516r (17). The thermocycling procedure for the second amplification was 94°C for 3 min, 35 cycles of 94°C for 30 s, 52°C for 1 min, 72°C for 1.5 min, and a final extension at 72°C for 10 min.

Regarding mycobiomes, nested PCRs targeting fungal 18S rRNA were similarly conducted using first-round PCR solution as a template and the fungi-specific primers nu-SSU-0817 and nu-SSU-1536 (8). Twenty-five microliters (final volume) of the PCR reaction solution contained 2.5 μL of 10× Ex Taq PCR buffer, 2.5 μL MgCl_2_ (25 μM), 0.5 μL dNTP (10 μM each), 1 μL of each primer (10 μM), 1 μL template, and 0.2 unit of Taq DNA polymerase (Fermentas, ThermoFisher). PCR was run under the following conditions: 35 cycles of denaturation at 94°C for 30 s, annealing at 52.5°C for 45 s, extension at 72°C for 1 min, preceded by 5 min of denaturation at 94°C and followed by a 10-min extension at 72°C. In each sample, triplicate PCR products were pooled and purified with a Tian Quick Midi purification kit (Tiangen, Beijing, China) in order to minimize PCR biases in cloning.

Clone libraries were constructed using the InsTAclone PCR cloning kit (ThermoFisher) according to the manufacturer’s instructions. The resulting plasmids were transformed into *Escherichia coli* DH5α-competent cells (Tiangen). Approximately 100 and 150 clones of each library were randomly selected for eukaryote and fungi, respectively. The presence of the 18S rDNA insert in colonies was checked by PCR reamplification with the vector-specific primers M13F and M13R using a small aliquot of a culture as the template. PCR amplification products of fungal clones containing the right insert size were digested with 1 U of the restriction enzyme *Msp* I (Fermentas, ThermoFisher) at 37°C for 1 h. Clones that produced the same RFLP pattern (DNA fragments of the same size) were considered to be members of the same phylotype and 4 clones of each phylotype were randomly selected for subsequent sequencing (Sangon Biotech, Shanghai, China). Putatively chimeric sequences were identified using Bellerophon (31) and removed from the libraries before subsequent analyses.

The eukaryotic and fungal 18S rRNA gene sequences obtained in the present study have been deposited in the NCBI database under the accession numbers MG753800–MG753972 and MG711921– MG712280, respectively.

### Phylogenetic analyses

Operational taxonomic units (OTUs) for eukaryotes and fungi (specifically designated as FOTUs to distinguish them from those of eukaryotes) were defined at a cut-off of 97% sequence similarity. It is important to note that species-level rDNA differences vary among eukaryotic groups; it is unlikely that a given rDNA cut-off will satisfy all eukaryotic species. We selected the 97% OTU definition because it is moderately stringent, able to overcome the major intragenomic variations of microeukaryotes (11, 23), and, hence, is more practical in ecological surveys. Representative sequences of each OTU and FOTU were selected using Mothur v. 1.39.5 (55). The higher classification of these representative sequences was initially made using the PR_2_ database v. 4.62 (26), and further checked by BLASTing against GenBank databases. A rarefaction analysis of clone libraries was performed by plotting the number of phylotypes detected against the number of sequences using aRarefactWin software (S. Holland, University of Georgia).

Phylogenetic analyses were performed in order to classify gut microbes to lower taxonomic ranks. The closest sequences of well-identified eukaryotic and fungal species were retrieved from GenBank and aligned with the newly obtained sequences using MAFFT v. 7.310 (35). Maximum likelihood (ML) and Bayesian trees were built using RaxML v. 8.0 (58) with the GAMMAI Model and MrBayes v. 3.2.6 program (54), respectively. Bayesian inference was executed with parameters set to four runs and four MCMC heated chains, a chain length of 3 million with a subsampling frequency of 1000, and a burn-in of 100,000. The chains were heated to 0.25 and a stop value of 0.01 was used.

## Results

### Detection of eukaryotic phylotypes in guts of the peanut and spoon worms

In order to detect gut eukaryotes in two marine worm species, 301 clones of 6 clone libraries were screened and sequenced. After discarding the 18S rRNA genes of the host species and 8 chimeric sequences, 173 eukaryotic sequences remained. Since the eukaryotic library for the spoon worm’s rectum (UU-r) yielded no valid sequences, it was excluded from subsequent analyses.

**Fig. 1:**
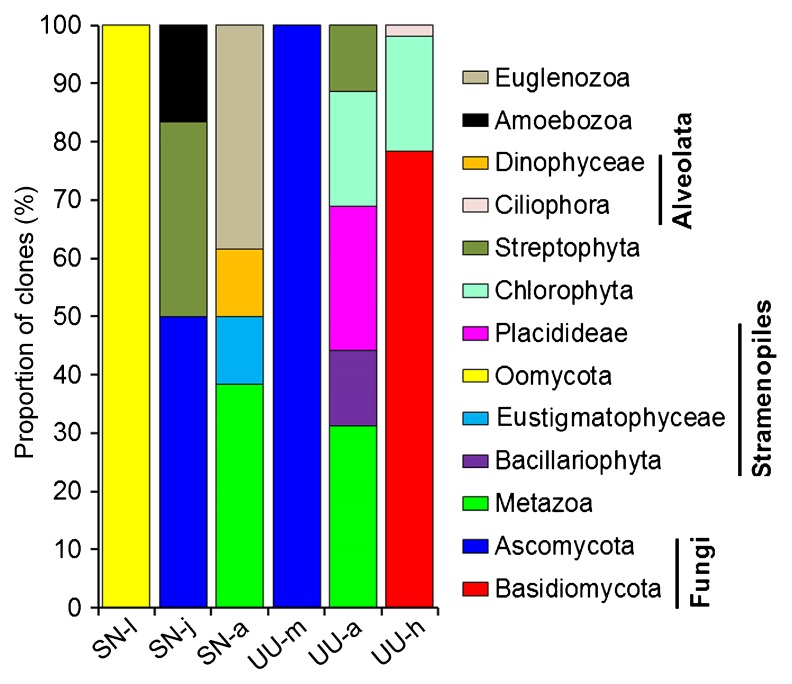
Variations in proportions of clones of eukaryotic groups based on a clone library analysis.

**Fig. 2:**
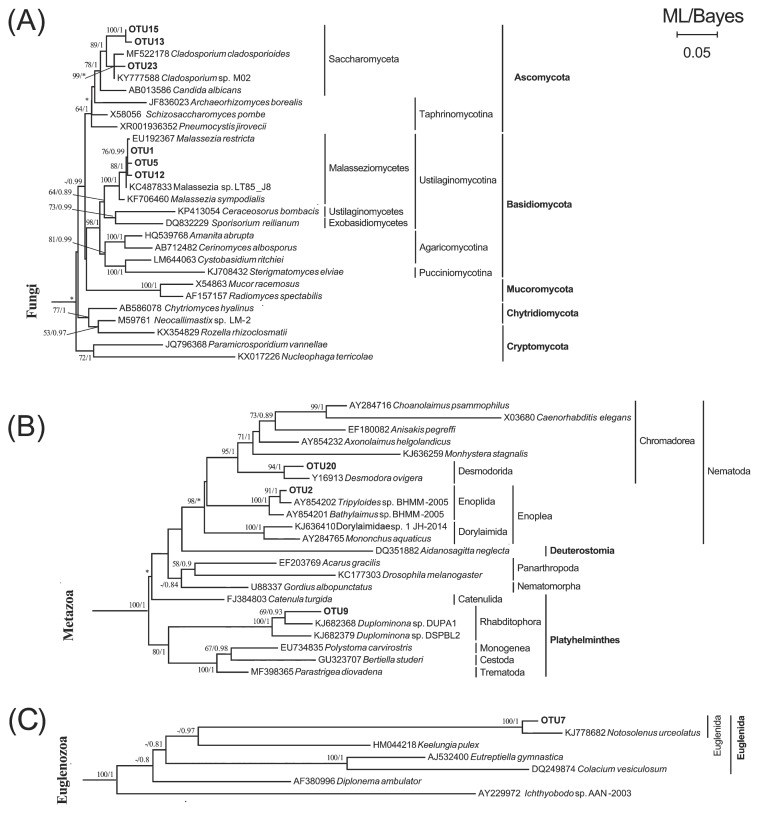
Maximum likelihood (ML) trees with a similar topology of the Bayesian tree based on 18S rRNA genes, showing the systematic positions of dietary eukaryotic OTUs detected in guts of *S. nudus* and *U. unicinctus*. Major clades of Fungi (A), Metazoa (B), and Euglenozoa (C) are presented. Asterisks indicate different topologies from the Bayesian trees. Only nodal supports with bootstrap values >50%, Bayesian posterior probability >0.8 are shown.

Twenty-five eukaryotic OTUs were obtained for the two worms (Table 1). The OTU-level composition of gut eukaryotes markedly varied among the development stages of *S. nudus* or across gut sections in *U. unicinctus*. BLASTing against GenBank showed that gut fungi had 6 OTUs, representing the most diverse eukaryotic group in the guts of these two animals. In terms of the proportion of reads, Fungi was the most abundant (28.9%), followed by Stramenopiles (26%), Archaeplastida (20.2%), and Metazoa (16.8%) and a few Euglenozoa (5.9%) and Amoebozoa (1.7%, Fig. 1 and Table 1). The results of the phylogenetic analysis indicated that half of these OTUs were closely related to members of the genus *Malassezia* and the remaining fungal OTUs belonged to Saccharomyceta (Ascomycota) (Fig. 2A).

Metazoan phylotypes were detected in both worms. There were 3 metazoan OTUs (Table 1), of which OTU2 was a member of the order Enoplida (Nematoda) and only detected in the aspiratory intestines of the spoon worm. Another nematode (OTU20) belonging to the order Desmodorida, together with a rhabditophoran flatworm (OTU9), were detected in the adults of *S. nudus* (Fig. 2B).

**Fig. 3:**
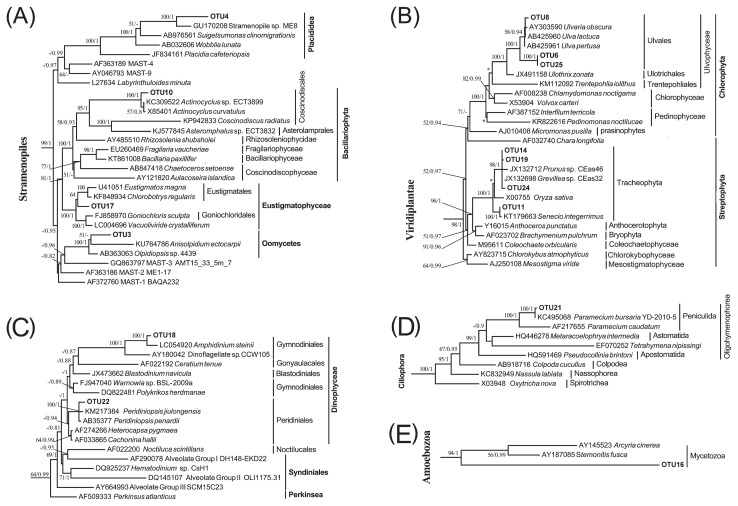
Maximum likelihood (ML) trees with a similar topology of the Bayesian tree based on 18S rRNA genes, showing systematic positions of dietary eukaryotic OTUs detected in guts of *S. nudus* and *U. unicinctus*. Major clades of Stramenopiles (A), Viridiplantae (B), Alveolata (C–D), and Amoebozoa (E) are depicted. Asterisks indicate different topologies from the Bayesian trees. Only nodal supports with bootstrap values >50%, Bayesian posterior probability >0.8 are shown.

Unicellular eukaryotes (protists) including autotrophs and heterotrophs were detected in the animal guts. A single phylotype closely related to *Notosolenus* (Euglenida) was also present in adult *S. nudus* (Fig. 2C and Table 1). An *Actinocyclus*-like diatom (OTU10) and placidid nanoflagellate (OTU4) were present in the aspiratory intestine samples of *U. unicinctus*. A phototrophic microalga, represented by OTU17 and affiliated with Eustigmatophyceae (Stramenopiles), was detected in adult *S. nudus*. A single eukaryotic phylotype (OTU3), which represented Oomycota (Stramenopiles), was exclusively detected in the larval peanut worm (Fig. 3A and Table 1). Furthermore, two OTUs of *Amphidinium* and *Peridiniopsis* dinoflagellates (Fig. 3C) and one OTU of Amoebozoa (Fig. 3E) were observed in the sediment-dwelling stages of *S. nudus*; one OTU of a *Paramecium* ciliate was detected in the hindgut of *U. unicinctus* only (Fig. 3D).

The detritus of higher plants appeared to be ingested by these two deposit-feeding animals. Four streptophyte OTUs were found to be affiliated with the phylum Tracheophyta (vascular plants) (Fig. 3B). Among these, OTU11 was closely related to *Lygodesmia juncea* and observed in the aspiratory intestines of *U. unicinctus* (Table 1), whereas the remaining 3 OTUs (OTU14, OTU19, and OTU24) were *Grevillea*-like and only found in juvenile *S. nudus*. Three OTUs of Chlorophyta were detected in the aspiratory intestines and hindgut and showed higher sequence similarities (97–99%) with *Ulva* spp. (Table 1 and Fig. 3B), indicating that spoon worms feed on these macroalgae.

Structural variations in gut eukaryotic phylotypes were evident across the growth stages of peanut worms. The most abundant group changed from Oomycota (100%) at the larval stage, to saccharomycetous fungi (50%) at the juvenile stage, then to metazoans (38%) and euglenids (38%) at the adult stage (Fig. 1). Fungal groups that dominated the gut eukaryotic phylotypes were abundant in the midgut and hindgut of spoon worms (Fig. 1).

### Composition of fungal communities associated with two marine worms

Seven clone libraries of 18S rRNA genes were constructed for gut fungal communities based on fungus-specific primers and PCR amplifications. A total of 1,005 clones were screened, 95 types were identified using an RFLP analysis, and 360 sequences were obtained after removing possible chimeras. At a cut-off of 97% sequence similarity, 48 FOTUs were identified (Table 2). Calculations of library coverage and rarefaction curves demonstrated that the sampling effort was sufficient to recover the most fungal phylotypes in these gut samples (Table S1 and Fig. S1). All FOTUs were affiliated with the subkingdom Dikarya, with 31 FOTUs of Basidiomycota and 17 FOTUs of Ascomycota. Basidiomycetous phylotypes were present in all 7 clone libraries, whereas ascomycetous phylotypes were absent in SN-j and UU-r.

**Fig. 4:**
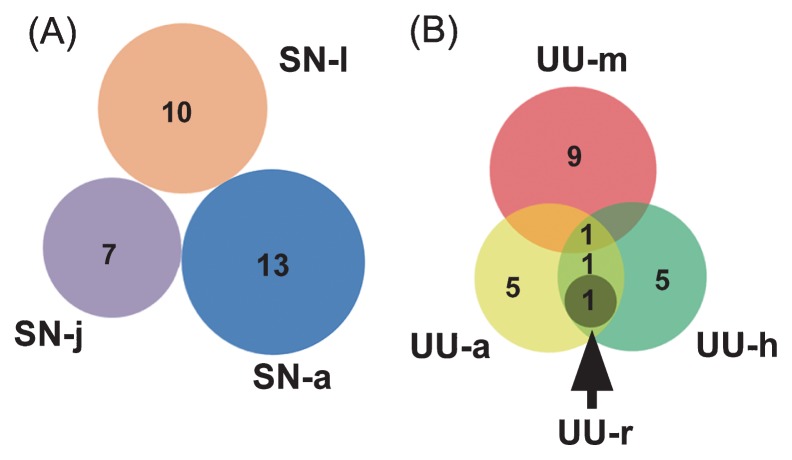
Venn diagrams showing numbers of fungal operational taxonomic units (OTUs) shared between clone libraries among life stages of *S. nudus* (A) and among gut regions in *U. unicinctus* (B).

Gut fungi shared no common OTUs among the different growth stages of peanut worms. Nevertheless, two FOTUs (FOTU1 and FOTU2) were consistently detected in 3 out of the 4 gut sections of spoon worms (Table 2). In the gut of *S. nudus*, FOTU richness varied slightly; it was the highest in the adult (13) and the lowest in the juvenile (7) (Fig. 4A). FOTU richness among different gut sections of *U. unicinctus* varied widely, with markedly fewer FOTUs (1) in the rectum than in the midgut (10), aspiratory intestines (8), and hindgut (8) (Fig. 4B).

**Fig. 5:**
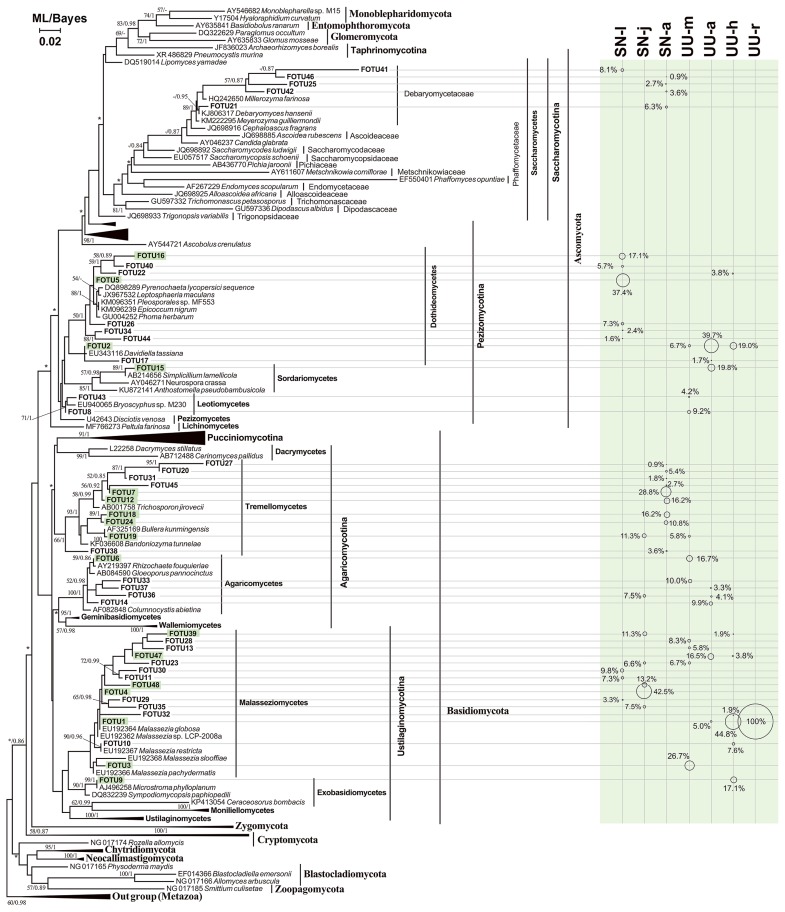
The maximum likelihood (ML) tree with a similar topology of the Bayesian tree, showing positions of FOTUs obtained from fungal DNA-targeted clone libraries. Asterisks indicated different topologies observed from the ML and Bayesian trees. Only nodal supports with bootstrap values >50%, Bayesian posterior probability >0.8 are shown. The size of the circle scales with the proportion (in percentage) of the FOTU in the fungal clone library.

By BLASTing the representative sequences against GenBank, 18 out of the 48 FOTUs were found to have <97% sequence similarities with the described fungal species (Table 2), indicating that the guts of these marine worms host many fungal species yet to be described. This was the case for the class Malasseziomycetes, 7 FOTUs of which were potentially new (Table 2). A phylogenetic analysis of these FOTUs resolved their systematic placements at lower ranks with reasonable confidence (Fig. 5). Overall, the gut fungi of these two marine animals were mainly represented by the classes Malasseziomycetes (15 FOTUs), Tremellomycetes (10 FOTUs), and Dothideomycetes (9 FOTUs). The classes Agaricomycetes, Agaricomycetes, and Saccharomycetes each had 5 FOTUs, whereas only 1–2 FOTUs were affiliated with Exobasidiomycetes, Sordariomycetes, or Leotiomycetes (Fig. 5 and Table 2). A large proportion of fungal phylotypes (52.5% clones) were yeasts closely related with Malassezia spp., *Trichosporon cutaneum*, *T. sporotrichoides*, *Debaryomyces hanseni*, *Cryptococcus zeae*, *Pichia sorbitophila*, *Cladosporium* sp., *Bullera alba*, and *Bandoniozyma tunnelae* (Table 2). These yeast-like FOTUs were mainly from Malasseziomycetes (62.5%), Tremellomycetes (30.6%), and Saccharomycetes (6.4%).

**Fig. 6:**
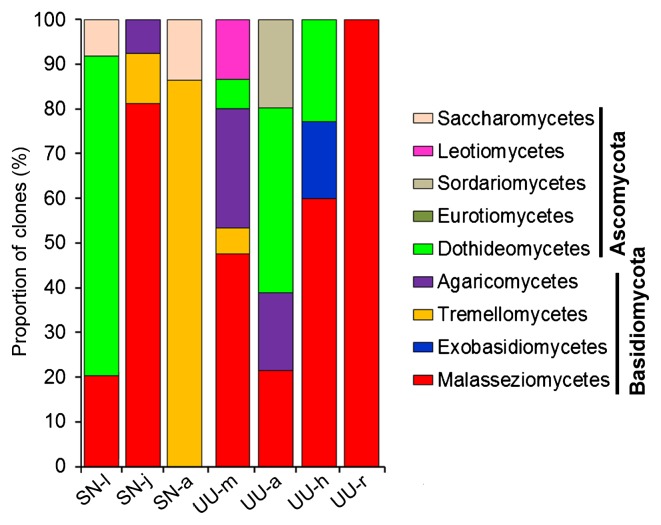
Variations in proportions of clones of mycobiome compositions based on a clone library analysis.

Fungal community structures in the guts of the two animals were characterized based on the clone numbers of the FOTUs (Fig. 6). Overall, the phylotypes of Basidiomycota (67.2%) appeared to be more abundant than those of Ascomycota (32.8%, Fig. 6). At the class level, Malasseziomycetes (40.1%) were also the most abundant, followed by Dothideomycetes (23.7%), Tremellomycetes (16.1%), and Agaricomycetes (8.5%). The other groups in the gut fungal communities were minor (<4%) (Fig. 6). Malasseziomycetous phylotypes were presented in 6 libraries, except for adult peanut worms. Saccharomycetous phylotypes were detected in *S. nudus*, but not in *U. unicinctus*. In contrast, Exobasidiomycetes, Sordariomycetes, and Leotiomycetes were only present in *U. unicinctus* (Fig. 6 and Table 2).

Across the larval, juvenile, and adult stages of *S. nudus*, the most abundant group in the gut fungal community shifted from Dothideomycetes (71.5%) to Malasseziomycetes (81.1%) and Tremellomycetes (93.2%). Nevertheless, in the 4 gut sections of *U. unicinctus*, Malasseziomycetes was consistently the most abundant (midgut, 47.5%; hindgut, 60.0%; rectum 100%), except for in the aspiratory intestines (21.5%).

## Discussion

### Eukaryotic diets in larval, juvenile, and adult stages of S. nudus and different gut sections of *U. unicinctus*

To the best of our knowledge, the present study is the first to reveal eukaryotic diet compositions in coastal sediment-dwelling sipunculan and annelid animals using molecular approaches. We detected a wide range of eukaryotic groups associated with the larvae of *S. nudus* and in the guts of both worms. The diets detected in juvenile and adult *S. nudus* included benthic microalgae (microphytobenthos) and macrophytes, which is consistent with previous findings obtained using a microscopic gut analysis (61). Nevertheless, our study using an 18S rRNA gene-based clone library analysis provided higher taxonomic resolutions to these prey in the peanut worm, which contributed to the interpretation of the trophic function of the predator. For example, dinoflagellates closely related to *Amphidinium steinii* and *Peridiniopsis*, eustigmatophytes, and *Notosolenus*-like euglenids were detected (Table 1). *Amphidinium* and *Notosolenus* species have both been commonly observed in marine sediments (2), and *Peridiniopsis* spp. are members of freshwater plankton (63). These findings provide evidence that peanut worms feed not only on benthic, but also planktonic microalgae, even including some from freshwater run-off, at sediment-dwelling stages. Eustigmatophytes are yellow-green algae, typically with small (<30 μm) cell sizes and abundant amounts of polyunsaturated fatty acids; some cultured species of this class are commonly used as food for larvae in mariculture (19), which was the case in the present study. The presence of eustigmatophytes in the gut of adult *S. nudus* implies that these small algae are still a valuable food source for the worm in its natural habitat. Sequences of *Grevillea* sp., a flowering tree mainly distributed in tropical and subtropical areas of China, were detected in the gut of juvenile *S. nudus*, indicating the ingredients of the artificial diet supplied during the pond culture period.

The observation of marked changes in eukaryotic diets at separate life stages of *S. nudus* is intuitive and straightforward because of the contrasting lifestyles (planktonic vs. benthic) and feeding modes (filtering vs. suspension+surface deposit-feeding) across the larval, juvenile, and adult stages of this species, as well as aquaculture management during the first two stages. Nevertheless, our molecular analysis revealed some novel aspects on what and how diets change. An important point is that *Anisolpidium* and *Olpidiopsis*-related oomycetes (pseudofungi) were detected exclusively at the planktonic pelagosphera stage. Oomycete phylotypes were also found in the larvae of the red rock lobster (50). *Anisolpidium* and *Olpidiopsis* species were brown and red algae-infecting pathogens that have the potential to cause great economic losses in the seaweed-farming industry (21, 37). Therefore, our results suggest that the flagellated zoospores of these oomycete pathogens may be filtered and ingested by the larvae of *S. nudus*, a potential trophic link between the macroalgal pathogens and planktonic larvae of cultivated animals, which may provide an insight into reducing the risk of an outbreak of oomycete diseases in seaweed farming. None of the microalgae (*i.e.*, 3 chlorophytes, 3 chrysophytes, and 3 diatoms) or baker’s yeast, which are good prey and generally applied for the larvae of *S. nudus* (40), were detected in the present study. These artificially prepared diets may have been easily digested or egested due to the short gut passage time in small-sized larvae. Apart from protists, higher sequence proportions of fungi and animals (*i.e.*, nematodes and flatworms) were found in the clone libraries for adult *S. nudus* and *U. unicinctus*, indicating a markedly broader food spectrum and the omnivorous nature of these two suspension-feeding worms.

The burrow-dwelling species *U. unicinctus* uses a mucus net to filter food particles in the water, and, thus, is a typical suspension or filtering feeder. It is commonly considered to be a non-selective feeder, whereas microscopic observation-based information on the gut content of the spoon worm has not been available (56). The present study is the first to characterize its gut contents. Our molecular analysis of midgut, aspiratory intestine, and hindgut samples collectively showed that the diet included the enoplid nematodes, *Actinocyclus* diatoms, of *Ulva* seaweeds, detritus of *L. juncea* (a typical sand dune-inhabiting plant near the coastal sites of sampling), and flagellates of the stramenopile class Placididea, indicating the omnivory of this worm. Our detection of *Ulva* species in the gut content samples of *U. unicinctus* collected at Yantai suggests that spoon worms feed on seaweed detritus. This trophic relationship is easily explained by the green-tide species *Ulva* frequently blooming in the summer at the Yellow Sea in recent years (64), resulting in some seaweed detritus drifting to and being buried in the intertidal zones of the coastal line of Yantai and then being consumed. This finding supports the food value of macroalgae to the sediment-dwelling macrofaunal community (*e.g.*, 41).

The described placidids are all heterotrophic nanoflagellate (HNFs), of which the bacterivorous species *Suigetsumonas clinomigrationis* inhabits anoxic environments (39, 51), suggesting that the placidid phylotypes identified in the present study are sediment-dwelling, distributed in the pore water of sediments, pumped into the burrow by peristaltic contractions, intercepted by the mucus net, and eventually ingested by *U. unicinctus*. Approximately 30% of the clones in the library of the aspiratory gut were from placidid HNFs, which contrasts with the rarity of this group in coastal and marine benthic microeukaryotes (24, 66), suggesting that placidid HNFs were selectively filtered and predated. Alternatively, placidid flagellates may be commensal, adapted to the anoxic gut environment. Further investigations are needed in order to clarify this issue.

During gut passage from the aspiratory gut to the hindgut, most protists and metazoan diets decrease, whereas *Ulva* and some basidiomycetous fungi remain, highlighting the high digestive efficiency of this worm and the residential nature of many fungal phylotypes in the animal’s gut. Fungal phylotypes appear to be important because they dominated the two libraries of eukaryotic diets in peanut and spoon worms in the present study. The dominance of fungi in eukaryotic diets has also been reported in other marine animals, *e.g.*, the anterior caecum of the irregular sea urchin (60) and the larval midgut of the red rock lobster (50). Nevertheless, it is still premature to examine the function of these gut fungi in order to establish whether they are ingested, inactive, commensal, or parasites (50).

### Varying gut mycobiomes at larval, juvenile, and adult stages of the peanut worm

The variation patterns of fungal diversity and composition across time and space need to be clarified in order to obtain a better understanding of their functions in the gut. The high coverage (≥97%) of all fungal clone libraries and rarefaction curves indicate that most fungal phylotypes have been recovered, which allows for alpha diversity comparisons of these fungal communities. Among the three stages of *S. nudus*, adults took a longer time to develop and had the longest alimentary tract, which enabled more fungal species to colonize, resulting in the highest gut FOTU richness. At the larval, juvenile, and adult stages, the fungal community was dominated by *Epicoccum nigrum*, *Malassezia* spp., and *Trichosporon-*related phytotypes, respectively. *Epicoccum* fungi are saprophytic and capable of producing antifungal and antibacterial compounds (9). It currently remains unclear whether the exclusive presence of these phylotypes plays a role in protecting larvae from other microbial pathogens.

*Malassezia*-like fungi are ecologically diverse and wide-spread yeasts (4), but not a numerically abundant group in the fungal communities of coastal water and sediment environments (27, 62, 66). In the present study, *Malassezia* spp. were prevalent in the juveniles and adults of these worms, indicating that this group of fungi are specifically enriched in the gut and, hence, appear to be residents rather than ingested transients. This group has also been detected in the guts of lobster larvae and Japanese eels (50, 59). In gut samples in which metazoans were absent and the sequence proportions of macrophyte and macroalgal phylotypes were high in eukaryotic diets (*e.g.*, the juveniles of *S. nudus* and the hindgut of *U. unicinctus*), there were high proportions of *Malassezia-*like phylotypes in the fungal communities. The proportion of *Malassezia*-like phylotypes generally increased during passage through the gut of *U. unicinctus*. These results suggest a relationship between *Malassezia*-like fungi and ingested plant detritus in the guts of sediment-dwelling worms. In metazoan-eating adults, there were representatives of *D. hansenii*, a saccharomycetous fungus with strong protease and lipase activities that contribute to digestion in a carnivorous diet (52).

*Trichosporon* spp. are basidiomycetous yeast-like fungi predominantly found in tropical and temperate areas and in the guts of humans and fishes (15, 28, 53). These fungi are capable of degrading amino acids, betaine, and aromatic compounds (3, 7). The dominance of *Trichosporon* spp. in the gut of tropical, adult specimens of *S. nudus* may be related to their thermal adaptation. Furthermore, a longer gut in the adult allows for the efficient digestion of food, leaving a higher proportion of refractory organic carbon in the gut content, which may have promoted the relative abundance of *Trichosporon*.

### Different mycobiomes in gut sections of the spoon worm

The composition of gut fungal community varied along the gut passage through the midgut, aspiratory intestines, hindgut, and rectum in *U. unicinctus*, exhibiting distinctness in each of these gut sections. Apart from the prevalence of *Malassezia-*like species, agaricomycetous *Climacodon* and *Rhizochaete-*like fungi were abundant in the midgut, whereas *Ceriporiopsis* sp. was abundant in the aspiratory gut. All three agaricomycetous groups are wood-decaying fungi that parasitize a range of trees and have seldom been reported in marine environments (14). The white-rot fungus *Ceriporiopsis subvermispora* preferentially removes lignin with relatively little cellulose degradation (20). Lignins are generally more difficult to decompose than celluloses, and from a functional point of view, it is logical to expect celluloses to be degraded by the mycobiome earlier than lignins during passage through the gut of the spoon worm; therefore, the lignin-selective *Ceriporiopsis* species occurred in a posterior part (*e.g.*, aspiratory intestines) of the digestive tract. Nevertheless, it currently remains unclear whether the presence of these fungi in the gut environment is functionally independent due to their spores associating with the tree detritus ingested by the animals.

Many *Cladosporium* phylotypes were detected in the aspiratory intestines and hindgut of *S. nudus*. *Cladosporium* species are cosmopolitan and frequently recorded in gut environments, such as in humans (25) and earthworms (10). Members of this genus are capable of degrading cellulose and lignin (38). Although less abundant than *Malassezia* and *Cladosporium*-related phylotypes, the exobasidiomycetous *Pseudomicrostroma glucosiphilum* also appeared to be numerically important in the hindgut of *S. nudus*. This species was originally isolated using a glucose air-exposed agar plate, and relevant ecological information is currently not available, except for physiological knowledge that it assimilates a range of carbon sources including cellobiose, trehalose, arabinose, and ribose and grows on vitamin-free medium (36). The present results add the gut environment as an additional ecological niche of this fungus.

## Supplementary Material



## Figures and Tables

**Table 1:** Classification and distribution of gut eukaryotic OTUs in libraries and results of BLASTing against GenBank.

Classification	OTU ID	SN-l	SN-j	SN-a	UU-m	UU-a	UU-h	Closest relative (accession number)	Identity (%)
Fungi, Basidiomycota	OTU1	0	0	0	0	0	21	*Sclerotium* sp. (AF010303)	100
	OTU5	0	0	0	0	0	13	*Sclerotium* sp. (JX132785)	99
	OTU12	0	0	0	0	0	6	*Sclerotium* sp. (AF010303)	99
Fungi, Ascomycota	OTU13	0	5	0	0	0	0	*Archaeospora* sp. (KT923272)	99
	OTU15	0	4	0	0	0	0	*Archaeospora* sp. (KT923272)	100
	OTU23	0	0	0	1	0	0	*Cladosporium cladosporioides* (MF522178)	99
Metazoa	OTU2	0	0	0	0	19	0	*Tripyloides* sp. (AY854202)	96
	OTU9	0	0	9	0	0	0	*Duplominona* sp. (KJ682379)	89
	OTU20	0	0	1	0	0	0	*Desmodora ovigera* (Y16913)	94
Archaeplastida, Streptophyta	OTU11	0	0	0	0	7	0	*Lygodesmia juncea* (KT179674)	99
	OTU14	0	4	0	0	0	0	*Grevillea* sp. (JX132698)	100
	OTU19	0	1	0	0	0	0	*Grevillea* sp. (JX132698)	99
	OTU24	0	1	0	0	0	0	*Grevillea* sp. (JX132698)	99
Archaeplastida, Chlorophyta	OTU6	0	0	0	0	11	0	*Ulva pertusa* (AB425961)	97
	OTU8	0	0	0	0	0	10	*Ulva lactuca* (AB425960)	99
	OTU25	0	0	0	0	1	0	*Ulva pertusa* (AB425961)	97
Alveolata, Dinophyceae	OTU18	0	0	2	0	0	0	*Amphidinium steinii* (LC054921)	96
	OTU22	0	0	1	0	0	0	*Peridiniopsis penardii* (AB353771)	98
Alveolata, Ciliophora	OTU21	0	0	0	0	0	1	*Paramecium bursaria* (KC495068)	99
Stramenopiles, Bacillariophyta	OTU10	0	0	0	0	8	0	*Actinocyclus* sp. (KC309522)	99
Stramenopiles, Eustigmatophyceae	OTU17	0	0	3	0	0	0	*Pseudotetraedriella kamillae* (EF044311)	95
Stramenopiles, Oomycota	OTU3	16	0	0	0	0	0	*Olpidiopsis* sp. (AB363063)	93
Stramenopiles, Placididea	OTU4	0	0	0	0	15	0	*Suigetsumonas clinomigrationis* (AB976561)	87
Euglenozoa	OTU7	0	0	10	0	0	0	*Notosolenus urceolatus* (KJ778682)	89
Amoebozoa	OTU16	0	3	0	0	0	0	*Arcyria cinerea* (AY145523)	80

**Table 2:** Number of clones belonging to each FOTU in genetic libraries and phylogenetic affiliations of representative clone sequences obtained using BLASTing against GenBank

Classification	OTU ID	SN-l	SN-j	SN-a	UU-m	UU-a	UU-h	UU-r	Closest relative (accession number)	Identity (%)
Basidiomycota										
Malasseziomycetes	FOTU1	0	0	0	0	6	47	30	*Malassezia restricta* (EU192367)	100
	FOTU3	0	0	0	32	0	0	0	*Malassezia restricta* (EU192366)	99
	FOTU4	0	45	0	0	0	0	0	*Malassezia globosa* (EU192364)	99
	FOTU10	0	0	0	0	0	8	0	*Malassezia pachydermatis* (EU192367)	100
	FOTU11	9	0	0	0	0	0	0	*Malassezia globosa* (EU192364)	96
	FOTU13	0	0	0	7	0	0	0	*Malassezia pachydermatis* (EU192366)	95
	FOTU23	0	7	0	8	0	0	0	*Trichosporon sporotrichoides* (KF036721)	96
	FOTU28	0	0	0	10	0	0	0	*Malassezia restricta* (EU192367)	95
	FOTU29	4	0	0	0	0	0	0	*Malassezia globosa* (EU192364)	97
	FOTU30	12	0	0	0	0	0	0	*Malassezia globosa* (EU192364)	96
	FOTU32	0	0	0	0	0	2	0	*Malassezia globosa* (EU192364)	97
	FOTU35	0	8	0	0	0	0	0	*Malassezia globosa* (EU192364)	97
	FOTU39	0	12	0	0	0	2	0	*Cladosporium* sp. (KU512834)	96
	FOTU47	0	0	0	0	20	4	0	*Cladosporium* sp. (KU512834)	96
	FOTU48	0	14	0	0	0	0	0	*Malassezia globosa* (EU192364)	97
Exobasidiomycetes	FOTU9	0	0	0	0	0	18	0	*Pseudomicrostroma glucosiphilum* (KR912075)	100
Tremellomycetes	FOTU7	0	0	32	0	0	0	0	*Trichosporon cutaneum* (KF036712)	98
	FOTU12	0	0	18	0	0	0	0	*Trichosporon cutaneum* (KF036712)	98
	FOTU18	0	0	18	0	0	0	0	*Trichosporon sporotrichoides* (JN939434)	98
	FOTU19	0	12	0	7	0	0	0	*Cryptococcus zeae* (FJ153133)	100
	FOTU20	0	0	6	0	0	0	0	*Bullera alba* (AY313034)	94
	FOTU24	0	0	12	0	0	0	0	*Trichosporon sporotrichoides* (JN939434)	98
	FOTU27	0	0	1	0	0	0	0	*Sirobasidium* sp. (LC203430)	93
	FOTU31	0	0	2	0	0	0	0	*Trichosporon cutaneum* (KF036712)	96
	FOTU38	0	0	4	0	0	0	0	*Malassezia globosa* (EU192364)	97
	FOTU45	0	0	3	0	0	0	0	*Trichosporon cutaneum* (KF036712)	96
Agaricomycetes	FOTU6	0	0	0	20	0	0	0	*Climacodon septentrionalis* (AY705964)	99
	FOTU14	0	0	0	0	12	0	0	*Ceriporiopsis* sp. (EU670846)	99
	FOTU33	0	0	0	12	0	0	0	*Rhizochaete fouquieriae* (AY219397)	98
	FOTU36	0	8	0	0	5	0	0	*Ceriporiopsis* sp. (EU670846)	98
	FOTU37	0	0	0	0	4	0	0	*Ceriporiopsis* sp. (EU670846)	96
Ascomycota Dothideomycetes	FOTU2	0	0	0	8	48	20	0	*Cladosporium* sp. (KU512834)	100
	FOTU5	46	0	0	0	0	0	0	*Epicoccum nigrum* (KM096239)	98
	FOTU16	21	0	0	0	0	0	0	*Epicoccum nigrum* (KM096239)	95
	FOTU17	0	0	0	0	2	0	0	*Cladosporium* sp. (KU512834)	98
	FOTU22	0	0	0	0	0	4	0	*Epicoccum nigrum* (KM096239)	97
	FOTU26	9	0	0	0	0	0	0	*Epicoccum nigrum* (KM096239)	97
	FOTU34	3	0	0	0	0	0	0	*Epicoccum nigrum* (KM096239)	97
	FOTU44	2	0	0	0	0	0	0	*Malassezia restricta* (EU192367)	95
	FOTU40	7	0	0	0	0	0	0	*Epicoccum nigrum* (KM096239)	97
Sordariomycetes	FOTU15	0	0	0	0	24	0	0	*Simplicillium lanosoniveum* (KY075854)	99
Leotiomycetes	FOTU8	0	0	0	11	0	0	0	*Thelebolus microsporus* (KJ939313)	99
	FOTU43	0	0	0	5	0	0	0	*Thelebolus microsporus* (KJ939313)	99
Saccharomycetes	FOTU21	0	0	7	0	0	0	0	*Debaryomyces hansenii* (LC041126)	98
	FOTU25	0	0	3	0	0	0	0	*Bandoniozyma tunnelae* (KF036608)	94
	FOTU41	10	0	0	0	0	0	0	*Trichosporon cutaneum* (KF036712)	92
	FOTU42	0	0	4	0	0	0	0	*Pichia sorbitophila* (FO082054)	94
	FOTU46	0	0	1	0	0	0	0	*Peltula farinosa* (MF766273)	93
