# Supplementary material for: Molecular Detection of Eukaryotic Diets and Gut Mycobiomes in Two Marine Sediment-Dwelling Worms, Sipunculus nudus and Urechis unicinctus
Source: Microbes Environ. 2018 Sep 29;33(3):290–300. doi: 10.1264/jsme2.ME18065 (PMC6167119; doi:10.1264/jsme2.ME18065)
Supplement: Supplementary file 1 [file 33_290_s1.pdf]

Fig. S1. Rarefaction curves for total eukaryotic and fungal communities observed from clone libraries of different gut sampls of *S. nudus* and *U. unicinctus*.

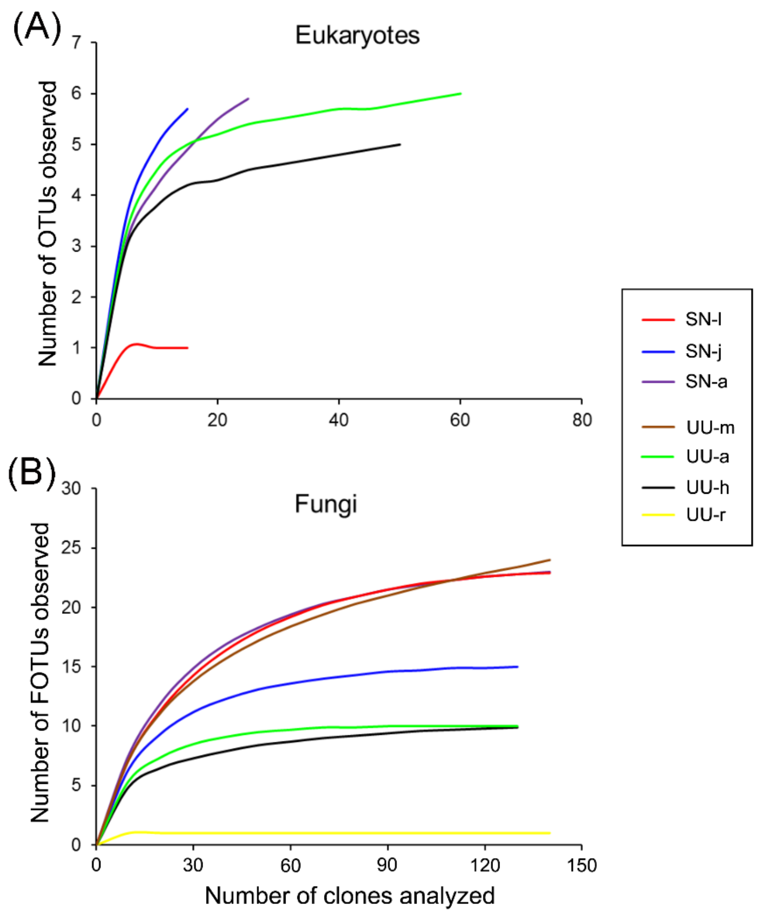

Table S1 Reads and alpha diversity indices derived from eukaryotic and fungal clone libraries of *Sipunculus nudus* and *Urechis unicinctus* gut samples.

	Eukaryotes							Fungi						
	SN-l	SN-j	SN-a	UU-m	UU-a	UU-h	UU-r	SN-l	SN-j	SN-a	UU-m	UU-a	UU-h	UU-r
No. of clean reads	16	18	26	1	61	51	0	123	106	111	120	121	105	30
Richness	1	6	6	1	6	5	-	10	7	13	10	8	8	1
Shannon	0	2.37	2.07	0	2.31	1.97	-	2.87	2.44	3.05	3.08	2.42	2.27	0
Simpson	0	0.79	0.71	0	0.78	0.71	-	0.81	0.76	0.84	0.86	0.76	0.72	0
PD	0.17	0.64	1.40	0.08	0.82	0.48	-	0.47	0.48	0.56	0.51	0.4	0.33	0
Coverage	1	0.96	0.96	0.96	0.98	0.98	-	1	1	0.98	1	1	1	0.97
